# Hearing the Unheard: Promoting Active and Healthy Aging With Family Leisure

**DOI:** 10.1093/geroni/igaa057.1111

**Published:** 2020-12-16

**Authors:** Tia Rogers-Jarrell, Brad Meisner

**Affiliations:** 1 York University, Mississauga, Ontario, Canada; 2 York University, North York, Ontario, Canada

## Abstract

Grandparents are playing an increasing role in the lives of their grandchildren. Participating in this role enriches grandparent’s well-being through feelings of enjoyment and satisfaction. Research also indicates participating in family leisure positively improves family communication, creates higher quality relationships, and enhances family cohesiveness and connectedness. However, the grandparent perspective, and their inclusion in family leisure, is often overlooked, rendering their voices invisible. Given the growing aging population, and the role grandparents play, their involvement in, and experience with, family leisure is becoming increasingly relevant. Therefore, the purpose of this paper was to critically review and explore the available literature on grandparents (ages 65+) experiences with family leisure. This paper critically reflected on benefits of family leisure participation according to the World Health Organizations active aging and healthy aging ideologies. Participation in family leisure provides grandparents with opportunities for social participation, remaining active and engaged through generativity and contribution to family, as well as building, maintaining and enhancing relationships. More research that is inclusive of grandparent’s perspective of, and experience with, family leisure is needed. By taking a socioecological approach to understanding the experiences of grandparents’ participation in family leisure, clear suggestions can be made on how to support grandparent’s involvement in family leisure at the interpersonal, organizational, institutional, community, and policy levels. In particular, future actions and initiatives should simultaneously support family leisure across and within these domains. Promoting family leisure is one way to support active and healthy aging not only among older adults, but among all individuals.

